# Transcending technology boundaries and maintaining sense of community in virtual mental health peer support: a qualitative study with service providers and users

**DOI:** 10.1186/s12913-024-10943-y

**Published:** 2024-04-24

**Authors:** Elmira Mirbahaeddin, Samia Chreim

**Affiliations:** Telfer School of Management, University of Ottawa, 55 Laurier Ave E, K1N 6N5 Ottawa, ON Canada

**Keywords:** Mental health, Peer support, Virtual services, Technology boundaries, Access boundaries, COVID-19, Sense of community, Qualitative research

## Abstract

**Background:**

This qualitative study explores the experiences of peer support workers (PSWs) and service users (or peers) during transition from in-person to virtual mental health services. During and following the COVID-19 pandemic, the need for accessible and community-based mental health support has become increasingly important. This research aims to understand how technological factors act as bridges and boundaries to mental health peer support services. In addition, the study explores whether and how a sense of community can be built or maintained among PSWs and peers in a virtual space when connections are mediated by technology. This research fills a gap in the literature by incorporating the perspectives of service users and underscores the potential of virtual peer support beyond pandemic conditions.

**Methods:**

Data collection was conducted from a community organization that offers mental health peer support services. Semi-structured interviews were conducted with 13 employees and 27 service users. Thematic analysis was employed to identify key themes and synthesize a comprehensive understanding.

**Results:**

The findings highlight the mental health peer support needs that were met through virtual services, the manifestation of technology-based boundaries and the steps taken to remove some of these boundaries, and the strategies employed by the organization and its members to establish and maintain a sense of community in a virtual environment marked by physical distancing and technology-mediated interrelations. The findings also reveal the importance of providing hybrid services consisting of a mixture of in person and virtual mental health support to reach a broad spectrum of service users.

**Conclusions:**

The study contributes to the ongoing efforts to enhance community mental health services and support in the virtual realm. It shows the importance of virtual peer support in situations where in-person support is not accessible. A hybrid model combining virtual and in-person mental health support services is recommended for better accessibility to mental health support services. Moreover, the importance of organizational support and of equitable resource allocation to overcome service boundaries are discussed.

**Supplementary Information:**

The online version contains supplementary material available at 10.1186/s12913-024-10943-y.

## Background

There is growing awareness around the world of the need to improve mental health services, yet the response to the need has been constrained [1]. The World Health Organization (WHO) has pointed to the urgent need to invest in community-based mental health services that prioritize a person-centred, recovery approach. Among these services, the WHO highlights the importance of peer support [1]. Formal mental health peer support refers to emotional and social support (Mental Health Commission of Canada, https://mentalhealthcommission.ca/what-we-do/access/peer-support/) provided by an individual referred to as a peer support worker (PSW). A mental health PSW is a person who has lived experience of mental health issues, has paid employment in a mental health support or services organization– often after receiving training– and offers intentional support to clients with mental health challenges through empathetic understanding and encouragement of self-determined recovery [2, 3].

Peer support is based on the belief that individuals who have navigated their own recovery experiences hold unique insights and lived practical knowledge that can be helpful in supporting others in their recovery (Mead, Hilton & Curtis, 2001). The notion of recovery in mental health refers to a multidimensional process that involves individuals actively engaging in their own well-being, making self-determined choices, fostering social connections, and pursuing a meaningful life despite the presence of mental health challenges (Mead, Hilton & Curtis, 2001). Peer support represents a political alternative to professionally led services and decision-making processes; it is an important approach for promoting the agency of individuals with mental health issues and reversing the power imbalances prevalent in the mental health system. Peer support can promote empowerment and self-efficacy, help enhance coping skills and strategies, and contribute to overall quality of life and emotional well-being [4–6]. It has been particularly helpful in situations where traditional professional mental health services might not fully address the needs of individuals or are not easily accessible [3, 7].

The importance of peer support became particularly salient during the COVID-19 pandemic. The pandemic adversely affected access to in-person mental health services, especially in jurisdictions where lockdowns were enacted. Peer support services in an online format created an opportunity to maintain availability and accessibility to basic yet important community-based mental health support [8]. A number of jurisdictions increased their peer support capacities by offering PSW training on remote services during the COVID-19 crisis (e.g., the Digital Peer Support Certification for peer specialists in the US that provided Medicaid-reimbursable virtual health services) [9]. Virtual peer support services have been beneficial in various ways including overcoming geographical barriers, reducing regional inequalities in access to providers, and offering convenience for a wide range of vulnerable populations in communities [10–11]. Hence virtual peer support has created *bridges* allowing people in need of mental health support to access it. These bridges can be advantageous not only in crisis situations such as the pandemic but also in non-crisis contexts by offering expanded accessibility.

There has been growing use of technology for a variety of mental health and support services with an aim to improve accessibility [10–12]. However, the move to provide mental health services and support remotely, despite its many benefits, also comes with challenges. These challenges include, among others, the need for providers and service users to adapt to the utilization of diverse technologies including synchronous (e.g. video calls) and asynchronous (e.g. apps) modalities [11]. We view the technological challenges as setting *boundaries* to providing, accessing and utilizing virtual services.

Existing literature does not provide adequate insight into how individuals adapt when a sudden and major change occurs from in-person to remote mental health and support services. Makarius & Larson (2017) state that the role of individuals in virtual work has been overlooked by considering them as “passive actors” [13, p.166] while portraying organizations as accountable for effective virtual work. They indicate that extant research on virtual work has tended to focus on virtual teams. Therefore, there is a need for a greater focus on individual experiences [14–15]. This applies in a general sense, but also, specifically to peer support. With the advent of COVID-19, PSWs became one of the forefront providers of mental health support [9]. Service users also had to adjust to virtual services. Yet limited knowledge exists about the individual experiences in the process of adapting and acclimating to using online mediums in virtual services in the context of peer support [16]. As virtual mental health services and supports are expected to continue to be used in the future, the experiences of individuals providing and receiving virtual peer support have become an important research topic.

Another issue of importance that needs to be considered when peer support is delivered virtually is whether technology-mediated connections allow peer support groups and individuals to maintain a sense of community. This sense of community is grounded in people’s relationship with a group that offers them membership, fulfillment of needs, and shared emotional connection [17], yet it is unclear whether the sense of community that is characteristic of in-person peer support is severed when services move online.

Earlier conceptualizations of communities emphasized the spatial dimension, defining communities as groups of people associated with a setting such as a neighbourhood or village [18]. McMillan and Chavis (1986) point to earlier work [19] that distinguished between the geographical notion of community (such as a neighbourhood or town) and the relational notion concerned with human relationships regardless of location [20]. McMillan and Chavis [20, p. 9] propose a definition of sense of community that applies to both of these conceptualizations, and is as follows: “Sense of community is a feeling that members have of belonging, a feeling that members matter to one another and to the group, and a shared faith that members’ needs will be met through their commitment to be together.” These authors point to four elements in their definition: (a) membership (a feeling of belonging or personal relatedness), (b) influence (a sense of mattering to the group), (c) integration and fulfillment of needs (a feeling that needs will be met through membership in the group), and (d) shared emotional connection (a belief that members have shared history and similar experiences) [20, p. 9].

In peer support communities, the principles of valuing individuals’ experiential knowledge of mental illnesses, determination for recovery, equality and reciprocity, and mutual agreement on what would be helpful for different individuals play a vital role [2–21]. People benefit in different ways by having a sense of community. They experience less isolation and social exclusion, have a greater sense of well-being, can call on support when they need it and learn from the experiences of other members [22–23]. Cronenwett & Norris (2009) examined the role of social collectives in providing peer support services to individuals with co-occurring disorders and the benefits of social support and shared experiences in promoting recovery [24]. However, it is not clear yet how peer support sense of community is created or maintained in situations where peer support moves to a virtual space and relationships are mediated by technological tools. To our knowledge, this topic has not been addressed despite its importance.

Given the importance of peer support and the recent surge in virtual peer support service provision, our objective is to understand how technological factors can act as bridges and boundaries to services, and whether and how a peer sense of community can be built or maintained in a virtual space that relies extensively on the use of technological tools. We aim to understand these issues from the perspective of individuals affected directly by the changes from in-person to virtual services. Therefore, we focus on PSWs who provide support services, and on the service users or clients– also known as peers. Inclusion of peer voices is particularly important, given that this is a gap in the literature since much research on peer support is based on the views of managers and PSWs, and not on the views of the peers themselves [25]. This limitation in the literature applies to peer support specifically, but also more broadly. For example, a systematic review investigating the implementation and adoption of telemental health found that research studies involved fewer service users compared to the number of providers (only 9 out of 45 included papers involved service users), indicating that the point of view of service users has not been adequately researched and little is reported about their experiences [26].

Hence, we ask the following research questions: What mental health peer support needs were met with virtual services? How were technology-based boundaries manifested and what bridges were built to open boundaries? How, if at all, was a sense of community established or maintained in a virtual space? We researched these topics in the case of a peer support organization that transitioned from in-person to virtual services during the COVID-19 pandemic. While in the case we studied the move to a virtual space was a response to exacerbated mental health challenges during the pandemic, it also opened up opportunities to understand if and how peer support could be enacted virtually *beyond pandemic conditions*. The surging interest in providing mental health services and support virtually thus makes our study a timely endeavor, and our findings a valuable addition to the literature.

## Methods

### Study design and context

We adopted an exploratory case study approach [27] as it allows us to understand complex social phenomena and generate new insights [28]. We aimed to achieve a deep understanding of how members of a peer support organization viewed or experienced mental health needs within the broader social context of the pandemic, how they interacted with technological aspects of virtual services, and the strategies they used to create a sense of community in a virtual space.

Our primary data consisted of semi-structured interviews with employees (PSWs and/or managers) and service users (or peers) of a peer support organization based in a major city in Ontario, Canada. This organization had more than twenty compensated PSWs, some of whom held managerial positions in the organization. It served the needs of a large number of peers who sought its various services. Before the COVID-19 pandemic, this organization primarily offered in-person services that included, among others, various peer groups as well as recreational and social programs which were also intended to provide support. We initiated the data collection in the early stages of the pandemic when lockdown regulations were implemented in Ontario. The reason for selecting this particular case was the organization’s rapid transition to virtual platforms in response to increased demand for peer support during lockdowns and isolation.

### Data collection

We collaborated with the organization in informing potential participants about the study. An email was sent by the organization to all its employees and service users informing them about the study, and inviting individuals interested in participating to contact the researchers. Thirteen PSWs and twenty-seven service users contacted the researchers. We interviewed all individuals who contacted us, thus our study included forty participants. Participants’ age ranged between being in their 20s and 60s, and the majority identified as female.

We conducted semi-structured interviews with participants. Different interview protocols were developed for each group of participants, and they were developed for this specific study. Based on the research questions and objectives, key themes were identified to guide the formulation of the interview questions. Moreover, the interview protocol was informed by existing literature on mental health peer support, the pandemic circumstances and concepts relating to boundary theory and sense of community. We adjusted the interview questions to account for feedback from the organization, whose approval we sought on the final interview protocols. A small group representing managers, PSWs and peers participated in providing feedback and validating the interview protocols. Overall, the questions were crafted to be clear and open-ended to encourage detailed responses and in-depth exploration of the subject matter. The interview protocols included questions on individuals’ mental health experiences during the pandemic, their experiences associated with opportunities and challenges of virtual services technology, the strategies that they and the organization used to capitalize on opportunities, remove difficulties, and build or maintain a sense of community. Open-ended questions enabled us to probe for additional details and allowed the participants to share beyond our questions, which provided us with rich and nuanced data [29]. The interviews were conducted via Zoom or phone, based on the participant’s preference. The interviews were conducted during the pandemic from February to November 2021. They were recorded and transcribed verbatim.

### Data analysis

We conducted thematic analysis and used the N-Vivo software for data coding and retrieval. Specifically, we followed the steps outlined by Braun and Clarke (2006) [30]. Familiarization with the data started with both authors conducting a number of interviews conjointly, taking notes during this process and discussing the preliminary data. Familiarization was enhanced by the first author’s transcription of the interviews. We then generated initial codes by immersing ourselves in the data. The long list of initial codes– or descriptive codes [28]– was closely related to participants’ words. We then identified emergent themes by grouping similar codes together and reviewing that the coded extracts fit the themes. The process involved constant comparison and was iterative in that we reviewed the codes and themes and changed the theme names when we identified emergent ideas based on new data. Analysis was mostly inductive, but we had also been sensitized by extant literature. In the later stage of the analysis, we grouped the themes into more abstract categories, continuously reviewing and refining the categories. Our final descriptive codes and theme list is presented in Table 1.

The first author performed the primary analysis and the second author reviewed the analysis on the basis of the data. When the authors’ interpretations differed, they returned to the data to find answers. This process offered confidence that the analysis was well anchored in the data from participants. We conducted member checking—explained in the next section— by seeking feedback from the participants on our analysis.

**Table 1:** Virtual mental health support services

Themes	Sub-themes	Descriptive codes
Need for Virtual Mental Health Support Services	*Boundaries related to accessing in-person services*: Increased need for mental health support and diminished access due to reduced in-person services	- Exacerbation of mental health issues and increased need for mental health support - Decreased access to mental health services due to persisting lockdowns
	Virtual peer support as a bridge to services	- Cost-effectiveness and convenience (free of charge, no wait time and inclusive) - Minimal logistical requirements to access - Improved accessibility for (a) peers with restricting illness or conditions, (b) new peers
Boundaries and Bridges relating to Telecommunication Technology for Virtual Mental Health Support	Virtual service technology boundaries	- Difficulty with technology equipment and connectivity - Telecommunication know-how/skillset - Some peers who previously connected in person did not join online services
	Virtual service bridges: Supports provided by the organization and PSWs	- Provision of technology & connection accessibility for providers and users - Delivering training and ongoing support - Exhibiting flexibility in operations & programs
Maintaining a Sense of Community in Virtual Mental Health Support Services	Maintaining continuous presence and social interaction	- Transitioning rapidly to providing virtual services - Setting up social integration opportunities/programs
	Establishing multiple points of connection	- Creating a variety of virtual programs - Adding a line of support via phone
	Building on organizational and peer culture	- Volunteering by peers - Leaning into peer values
	Acting collectively	- Making decisions collectively (minimal hierarchy) - Building capacity by pooling the staff’s expertise
	Sharing lived experiences and learning together	- Sharing and reciprocating feelings - Learning collectively through shared stories

### Establishing trustworthiness

We took several steps to establish the trustworthiness of the study [28, 31]. Two researchers worked together on data analysis, returning to the data when disagreements emerged. This offered triangulation through the involvement of two researchers. We also report extensive quotes from our participants as evidence of our analysis. In addition, we conducted member checking to determine whether our findings captured well the experiences of participants and thus ensured the credibility of the results. This entailed sharing a draft of the manuscript with the participants and asking them to provide their feedback on the researchers’ interpretation and whether those aligned with their experiences. We received feedback from two PSWs and five peers, all of whom were in agreement with the results reported. One participant commented, “*I feel that the paper captured… challenges and victories peer supporters experienced during COVID*” and another participant stated, “*It is a good in-depth work/story showing the mental health challenges and how those were addressed during the pandemic, how people evolved from their experience and stood for each other when it mattered the most.*”

### Research ethics

The study was approved by the Research Ethics Board (REB) of the University of Ottawa (Reference number S-11-20-6226). All study participants were fully informed about the project through both written and oral communication, and willingly gave their consent. The consent form included information about mental health resources available to them if needed, and participants were informed about their right to withdraw from the study. All procedures followed the appropriate guidelines and regulations.

## Results

We begin with the results showing the need for virtual mental health support during the pandemic and follow with the technology-based boundaries and bridges identified in virtual mental health support. In the last section of the results, we focus on the strategies that were used by the collective to maintain a sense of community despite the physical distances. It is important to note that we give attention to pandemic-related dynamics where pertinent, but also go beyond the pandemic context to address more general issues related to virtual peer support that were central in our participants’ accounts.

### Need for virtual mental health support services

#### Boundaries related to accessing in-person services

The pandemic amplified social issues that resulted in a surge in mental health challenges. Peers shared concerns regarding social vulnerabilities that became exacerbated during the pandemic. They told us about their challenges which included homelessness, domestic abuse, and struggles with addiction that were exacerbated during lockdowns. One peer referred to the “*downward spiral [of mental health] once the COVID-19 pandemic hit”.* A peer pointed out that *“literally everything shut down in the city…the needs of the community are just desperate*”, and a PSW stated that *“with the pandemic, there was a lot of isolation, and it was really hard…also just the transition back as things started opening up. It’s really anxiety provoking for a lot of people.*”

There was also difficulty finding mental health services as there were lengthy wait times to see a mental health professional. A peer stated: *“I think the most difficult thing was probably finding people to connect with…. There was a three-month waiting list to be able to even speak to anybody.”* It is important to note that accessing mental health services in person was difficult for many people even before and regardless of the pandemic. The following quote by a peer illustrates one of many situations under which accessing in-person peer support can be difficult: *“When you have a baby, it’s hard to be somewhere on time and remember to bring everything that you need and deal with the cranky baby… When your expectation is that you’re going to participate in these types of groups in-person, it can be very jarring*”.

#### Virtual peer support as a bridge

Virtual services can be a bridge connecting individuals to mental health peer support, especially when these individuals experience challenges with attending in-person peer activities. The peer who reflected above on the difficulties associated with accessing in-person peer support pointed out that “*when you can proceed in groups virtually, you can mute yourself, you can step away, your baby’s crib is right there…. So it was a really wonderful option.”* A peer reflecting on the high cost of seeking *“formal therapy”* and the inconvenience of doing so, pointed out that virtual peer support was *“a light in the tunnel”* for them: *“it was free, it was accessible, it was easier to find a peer support group during times that I could access it. During the pandemic, I accessed more groups than I did [in person].”*

We also heard from peers whose anxieties had been exacerbated during the pandemic. A peer shared that seeking in-person mental health support was a major challenge. This person added that “*it was nice to be able to access things from Zoom”.* Considering the risk of contracting the coronavirus, peers felt that not having to leave the house gave them a *“sense of accomplishment”* because accessing services remotely helped them remain engaged. A peer noted that virtual peer support had been “*the winter month survival*” for many individuals.

Peers also told us that virtual support was helpful for them in general, and not only because of the pandemic. Social anxieties, unrelated to the pandemic, were often mentioned by participants. A peer stated: *I’m very timid to talk in a support group, and with Zoom, I feel I can raise my hand with the computer and I get to speak. Whereas in a peer support group in person, I don’t always get to do that. And… you get to see everybody’s facial reactions when you’re in the gallery view [on Zoom], whereas you can’t do that when you’re in the group because I’m very shy and very anxious.”*

Anxieties were also related to driving. A peer stated, *“I feel grateful I don’t have to drive far or pay for parking. Without the anxiety of driving and being on time too is very relaxing… (Virtual peer support) is a blessing.”* For some individuals for whom transportation to in-person meetings could be difficult due to time or financial constraints, virtual services opened the possibility of receiving peer support.

Peers also told us how the virtual services facilitated receiving support in cases where struggles with depression kept them from seeking in-person services: “*If you’re so depressed, it’s hard to get out of bed… That’s another thing about Zoom, you don’t have to worry so much about your appearance. If you haven’t washed your hair that day, it’s fine… It makes it so much easier to attend*.”. Virtual services were also very helpful for peers who felt they needed to seek support frequently: “*I’ve struggled with feeling alone and… feeling overwhelmed… If I had to go to a walk-in, I wouldn’t have done it. I wouldn’t have had access and that would have been bad for me*.” Moreover, peers who felt self-conscious about their appearance, had experienced weight shaming, or physical differences found it more comfortable to attend virtual meetings because they *“take away the self-consciousness”* as a participant stated. By allowing participants to control what they reveal (e.g. by turning the camera on or off), virtual meetings may offer a certain sense of safety that in-person meetings may not provide.

Importantly, we were told that new members had joined virtual meetings who had not previously participated in in-person peer services. A manager pointed out that *“a lot of new people who were not previous members have joined the community to get support or to get social interaction”* and a PSW stated: “*we are supporting more people now. Our meetings are much larger. I’ve had people contact me from other provinces asking ‘Am I allowed to join?’ We’ve decided that as long as we have the capacity, anybody who wants can come*.”

In sum, virtual services offered benefits for individuals who struggled with various issues including anxieties and depression, or whose life circumstances made it difficult for them to commute to in-person meetings. Although the pandemic (and the lockdowns associated with it) exacerbated some of the challenges that people had faced, the quotes above indicate that some challenges were not specifically pandemic-related, but rather pertained to more general mental states and life circumstances. The fact that virtual meetings drew in attendance from individuals who had never been to in-person meetings is a further indication that virtual platforms increase accessibility for peers.

#### Boundaries and bridges relating to telecommunication technology for virtual mental health support

Accessing virtual services offered peers opportunities to receive support, but accessing these services had its own challenges. A major challenge was technology, which manifested in terms of access to and compatibility of devices, access to internet connection, and basic technological skills. We report on these challenges and on how they were mitigated.

#### Virtual service technology boundaries

Technology-based challenges were associated with access to and use of equipment, access to internet connections, and limited technology-based skills. Some individuals from both groups (peers and PSWs) found it difficult to transition to virtual services due to the unprecedented complexities introduced by the new service environment: “*the hardest thing for people is the technology part of it*.” The experience of change to virtual services was described as “*anxiety-provoking*” for people who were not familiar with the use of technology such as computers and smartphones in daily life.

Accessing virtual services required the use of the appropriate equipment such as smart phones, and for some peers, access to these devices was a challenge. A peer described: “*The devices that I had access to were lower-end devices… My cell phone was blocking out and freezing*”. Another peer stated: “*I would drop in occasionally using my phone. But I didn’t have a computer, and currently, I’m receiving disability benefits… As far as having money to burn, that’s not an option for me, it’s a very tight situation*”. In addition, lack of access to and reliable internet connection was another boundary. A participant described the lockdown situation: “*It was a big shock. It’s a big change. It’s forcing a lot of people who didn’t have the Internet to get Internet. So that caused a lot of stress and strain on a lot of people*”. Peers who shared an internet connection with multiple residents had to coordinate schedules since simultaneous Zoom calls could interrupt connections.

For some individuals, a lack of technology-based skills was a boundary. Some peers had difficulty navigating the nuances of the various platforms and their compatibility with the devices they were using: “*You had to figure out what platform was used and whether or not your technology was going to be compatible with it.*” Other peers experienced difficulties early on with logging in and accessing meetings: *“[It was a] struggle with the process of getting signed up, to get the notifications, to get the information*”. Others reported difficulty navigating the programs’ options during the meetings (e.g., using the raise hand option). The challenges did not only pertain to peers. PSWs also faced difficulties with technology: *“I did not have the technology needed to be able to do my job from home. I had a smartphone, but it’s still very challenging to host a Zoom group when I can only see 4 little faces on a screen.”*

#### Virtual service bridges: supports provided by the organization and PSWs

When the lockdowns were mandated, concern about peers’ mental health needs drove the organization to create a variety of platforms through which peer support services could be accessed. Within a few weeks, the organization created remote services to maintain continuity in support for peers. A PSW pointed out *“They were relying on us for their well-being.”* This created a sense of urgency to adapt quickly in order to meet the needs of the community.

Efforts were quickly deployed to connect with peers by phone and to create accessibility through online options. As a peer stated, they were “*trying to make things just as accessible as they could be*”. To this end, the organization engaged in advocacy efforts with external partners to provide devices, data, and internet connection to those without technology. A manager stated: *“Many people with mental health and addictions don’t even have access… We have been providing people with tech and tablets and smartphones and connectivity, and we’re a peer agency, we don’t have this kind of stuff!… I kept raising it at our (regional health authority) table with a lot of people who are very high up. And they said, ‘Let’s do it’! So we applied and put together a proposal… We now have contracts with [internet] providers, so [one company] provides the smartphones with sim cards and [another company] provides the tablets.”*

PSWs walked peers step by step through the Zoom functions that they needed in order to attend and participate in virtual meetings. A PSW pointed out: “*We did a lot of one-on-one training and coaching and mentoring with people to help them get their virtual equipment set up. At first, it was a lot of, ‘this is how you set up Zoom, this is how you set up your camera’… and then more people got comfortable using it*.”

PSWs also received training and support. Training included group and one-on-one sessions, and manuals were made available to provide instructions for an online environment: *“In the beginning, we had training from a staff member who is a certified online facilitator… and it walked us through how to use Zoom. I also had one-on-one training… to walk me individually before doing any online groups… I asked my questions, and felt comfortable then to roll with it, [and] manuals were written with the policies of how we were gonna do this online.”*

The social media team of the organization also became very active during the early days of the lockdowns. A manager who was part of this team described the role of the social media team: *“We re-did all the posters we had for in-person, we switched them to virtual, giving new contact information, laying out the registration process…Every day we posted what groups we had going on, and all of that content had been created after the pandemic started. Again, a lot of that very quick adaptation to the needs.”* We were also told that the organization added and adjusted online group activities and services as the lockdown policies and the needs of the peer community changed.

In sum, the findings show the challenges and solutions relating to using telecommunication technology for virtual mental health support during the COVID-19 pandemic. Accessing and providing these virtual services required access to and compatibility with devices, reliable internet connection, and technology-based skills, which could be challenging for some individuals. To address these challenging access boundaries, the organization arranged to provide devices, data, and internet connections, along with training and ongoing support to both peers and PSWs. Meanwhile, the organization also experienced a learning curve as it was adapting to the new circumstances and applied efforts to bridge the gaps in service access.

### Maintaining a sense of community in virtual mental health support services

The peer support community already existed before the pandemic lockdowns. Peers would come to the organization locale for in-person services and programs, and many relied on these programs for mental health support. The lockdowns were disruptive of the in-person programs, which had to be halted, and as we elaborated earlier, the organization quickly responded by establishing services online. We were interested in whether and how a sense of community could be re-established and maintained in a virtual environment. Our findings point to five strategies in which the organization and the peers engaged, and which enabled maintaining a sense of community. We present these strategies next, and would like to point out that although we discuss them separately to facilitate the presentation, these strategies were not mutually exclusive.

#### Maintaining continuous presence and social interaction

In a context of increasing isolation, and to meet the needs of peers, the organization quickly began to offer phone services whereby peers and PSWs could connect by phone. Participants told us the phone support communicated a sense of caring and had a significant impact on individuals’ mental health during the pandemic. One of several volunteer peers who took on the task of checking on other peers regularly, indicated that for some individuals, their only connection to the outside world was through these phone calls: “*It could mean the difference between being stable and unstable… Being unstable for a long time could lead to something terrible*.” Phone calls were not only about mental health topics, but could also include friendly conversations about daily living activities, which solidified relationships. The peers looked forward to these phone calls as a means of getting positive contact with someone who cared to listen. As one peer said, *“They opened up a phone line and… I would call almost every day… I really needed [peer support]… So having that as a service was really, really good.”* And another peer stated:*“[It was great] knowing that they’re always there. It’s just the comfort of knowing there’s someone to reach out to.*”

It is important to note the speed with which the organization was able to adapt and to create programs that met the peers’ needs, thus maintaining a continuous presence. As a manager stated, “*[peer support] works well in a pandemic because we were able to be more flexible.”* This is in contrast to institutional mental health services that were subject to various regulatory restrictions that would delay the introduction of online services. A PSW stated, “*we are extremely adaptable.”*

In short order, the organization created a variety of online groups and activities in which peers could register and participate. These programs allowed the peers to continue interacting and engaging with one another. The sense of community was palpable even for peers who did not participate actively in the programs: *“So for these people [like me], even though their videos and microphones are off, being immersed in the group, feeling like, hey, I’m not the only one, these are my people… and they look good and they’re talking and they’re feeling great. I feel good being there. And I may not want to say anything. It’s amazing. It’s a good feeling.”*

Another peer commented on the relationships with the PSWs in the virtual meetings and said “…*you can access [virtual support] anywhere and see the facilitators that you’re connected to. And that sometimes is enough to just make my spirit go fly.*” A similar sentiment was communicated by PSWs, one of whom stated: *“We have things seven days a week that peers can come and join us. That has been really great; [it] helps keep the sense of community because we have that touchpoint with them. “*

#### Establishing multiple points of connection

The organization was intent on meeting the diverse needs of peers, and to this end, created a variety of virtual programs and groups as well as phone services. In addition to the mental support groups, there were special activities such as yoga, crafting, and cooking, all of which instigated mutual support. These various activities could draw in diverse people who share similar interests, creating online communities. Peers stated that despite the lack of one-on-one eye contact, they found online groups were effective in offering valuable social activities related to wellness, nutrition, parenting, and gender-based support. One peer noted, “*They have a variety (of services)… Sometimes I’m in the mode of meeting [people], or joining arts and crafts. Sometimes I join the trivia online.”* Another peer indicated that it was possible *“to find the niche of the thing that you were looking for*” and a third peer stated: “*the trivia for me is very engaging… everybody can play.*”

The availability of multiple points of connection implied that the peers and PSWs could remain connected to each other on a regular basis. Another initiative by the organization to encourage this sense of community was the creation of a Facebook group. Due to the variety of points of contact, new members joined as they learned about the virtual services, expanding the community. However, the main aim of the organization remained to continue providing mental health support. A manager stated: *“A lot of what people wanted was social connection, which we do offer in recreation. But we’re a support-based organization, and even our recreation has some support components to it. We came up with this private Facebook group which has helped a lot with that because people can stay in touch, not just with facilitators or with a group in a moment, but they can talk to each other whenever they want should they choose to join. “*

#### Building on organizational and peer culture

Participants pointed out that peer culture is permeated by care and concern for members, and this was clear in various quotes we reported above from managers, PSWs and peers. In fact, managers and PSWs are also peers and they pointed this out continuously during our study. For example, a manager stated: “*It’s very helpful when peer support is informed by a community of people. And when peers can run some of their own services and see that peers are not only people who are recipients of services but actually are also managers*”. This manager also pointed out:*“A peer-run community of peer supporters can help people meet different needs: their creative needs, their social needs, their support needs. There are physical needs, we’re doing some walking. We’re supporting people to get technology so they can not only take part in our Zoom meetings but also order their own groceries online or maybe they can talk to their doctor online now. Peer support has a lot of strengths.”*

Another manager noted, “*It’s never just a job for people [at the organization]. It’s about how we can create something that is going to benefit the people who need it*.” This focus on helping and supporting each other was integral to the organization’s mission and culture. This focus was shared by peers. Increased involvement of peer volunteers, who were not paid by the organization, in running services including the voluntary phone line was highlighted as an example of peer values and practices. A manager explained, “*One of the things that’s really important is to rely on the people who are actually DOING the thing, as opposed to me saying “well I know what’s good for this”, but actually leaning into our values*.” Various participants mentioned that the implementation of online mental health support during the pandemic was an indication of resiliency in the peer support community. A peer stated “*We weren’t able to meet face to face. So people took it upon themselves to set up and organize these meetings and to learn how to use the technology to provide those services.*”

#### Acting collectively

The sense of community was also enabled by how decisions were made in the organization and with the help of peers. Deciding and acting collectively helped maintain a sense of community in the virtual space. This approach was especially effective during times of disruption that affected the organization and the peers. Overall, the organization’s collaborative approach to decision-making and focus on benefiting those in need were key components of its success.

The organization relied on discussion-based decision-making, with all staff members coming together weekly to discuss various issues and make decisions for the week. The management approach was collaborative and non-hierarchical. A manager said, “*We make decisions with the management collectively, and at times, when it’s appropriate, we make decisions with all staff*.” Another manager described how “*the hierarchy felt a lot flatter”* during the pandemic and the priority became *“Who’s got what competencies? Who’s got what skills? Bring them in!”*. Different members of the organization contributed their knowledge and skills to enhance the capacity to move services online. A PSW said: *“We all bring our own perspectives. So I said my specialty is looking at the programming and the scheduling and what is feasible for us as staff… it was a lot of communication.”*

#### Sharing lived experiences and learning together

Peer support is based on the shared lived experience of individuals. Sharing these experiences helps build bonds among peers. We were interested in how the virtual environment could have affected the sharing of experiences. Although some peers pointed out that they found it easier to share experiences in person, others– as we showed earlier– indicated that the online environment made it easier for them to participate. A PSW indicated: *“We offer that space to just connect… Even though we’re saying “You gotta raise your hand before you talk”– that was an adjustment period. But now it’s the norm… That sense of belonging comes from connecting around shared lived experiences. So connecting around that shared lived experience is still happening. It’s just virtual, and a little more systematic.”*

A peer described how the shared lived experience was helpful when using virtual services during the pandemic: *“The ability to participate with other people who are struggling [was helpful], I just think that sharing those feelings and hearing that you’re not alone was worthwhile to me*”. Another peer reflected on the importance of the virtual services for connection around shared experiences of feeling “lost”: *“It was a wonderful place to connect with people who were also struggling when everybody was sort of lost and in the same boat”.*

Shared experiences were not limited to feelings of being lost and struggling. Members were also learning together, which solidified the sense of community. A peer pointed out: *“[Relationships] became stronger in a sense, because we were all in the same boat… Sometimes the facilitators themselves were like I don’t know how to do that*. *We were all learning…and figuring things out. And I think that’s a good way to become closer to people.*”

In sum, various strategies were used by the organization and the collective (including PSWs and peers) to build and maintain a sense of community that was anchored in peer culture values.

### Continuation of mental health support through a hybrid mode: importance of combining in-person and virtual services

Virtual peer services were “a lifeline” especially during the pandemic, as a peer noted. However, some peers also looked forward to returning to in-person services for various reasons. For some, the in-person services provided structure to their week and a chance to leave the house. A peer noted: *“It forces me to get out of the house…I’m having difficulty leaving the house…half of me looks forward to it [the weekly support meeting], and half of me dreads it. But in the end, I get myself out of the door and I walk up to the center…I feel so much better afterwards.”*

Naturally occurring conversations during coffee breaks or after the meetings, which contribute to supporting relationships, were missed. As one peer stated, “*A lot of it [peer support] is the action piece and when you’re connecting virtually, it’s just not the same as being in person*”. Some participants pointed out that in-person interactions offered a deeper level of connection through shared energy and physical space. A participant noted, “*When someone’s super upset, you can feel it. When people are in their own homes, it feels disconnected because there are so many other people there. I feel like we’re seeing less emotional distress, whereas in-person, it would be brought out– and not distress in the sense that they’re not coping, but that they’re bringing big feelings or things on their mind and they’re expressing them freely in person. I feel there’s a lot less of that since being virtual*.” Additionally, some participants felt *“strange”* expressing strong emotions through a computer screen and pointed out that virtual settings offered less authentic connections compared to in-person interactions. Nonetheless, participants acknowledged that some people could still struggle regardless of the mode of interaction.

It was also pointed out that although virtual events drew in people who had never attended in person, some peers who used to attend in-person meetings did not join any virtual meetings, and it was not clear why this was the case or how they coped with the pandemic. Some of these individuals could not be found on online platforms to connect with. A participant stated, “…*there’s a whole voice of those who can’t access virtual, those who have only been going in-person… So I think we definitely should try to cater to both [when designing mental health support services*]”.

Overall, peers expressed support for maintaining remote online mental health peer support services even as lockdowns were lifted, and pointed out that transitioning to a hybrid mode would offer efficiency in resource utilization and greater convenience for remote access. A peer emphasizing the need to continue the virtual services noted the importance of social integration for peers with disabilities: “*I think there’s a lot of people, especially with disabilities or just more issues who have a really hard time going in person. I feel like there’s a lot more people who were able to access services and I don’t think that they should just be cut off and done.*” Those living on the outskirts of the city or with other commitments had limited time to attend in-person support meetings, making hybrid services desirable after pandemic restrictions were lifted. Online meetings made mental health services more accessible, allowing individuals to manage their work-life domains more harmoniously. A peer said: “…*People are always finding it a stress release and I like accessing it (peer support) from home sometimes instead of having to go to places…Sometimes I’m just not into seeing people, or going out and dealing with traffic.”*

In sum, continuing with virtual services while also maintaining in-person services was seen as offering more access to peer support services to a broader population, and as providing more choice for individuals who sought peer support.

## Discussion

This study contributes to the literature in a number of ways. It emphasizes the importance of providing virtual peer support in situations where mental health in-person support and services are not possible or accessible. We have highlighted the technology-based challenges and opportunities that create boundaries and bridges respectively to peer support in a virtual space. We have shown that a hybrid model involving both virtual and in-person services offers better accessibility to individuals and groups in need of support, and have argued for the importance of maintaining both modalities. We have also shown that a sense of community can be established in a virtual space, and have highlighted the strategies that peer organizations and their members can utilize to maintain the community spirit. As importantly, we have contributed to the literature by including peer voices and highlighting their experiences in their own words. Researchers have pointed out that the experiences of service users have not been adequately researched [26] and this is particularly so in the case of peers [25]. Our research enhances understanding of service users’ lived experiences.

### A hybrid model of peer support services

Our findings show, consistent with the literature, that each of virtual and in-person peer support service has its own advantages and disadvantages when used singularly, and that the joint operation of virtual and in-person services through a hybrid model provides more accessible service [32]. Using both approaches conjointly offers the opportunity to strengthen community-based mental health, and to reinforce recovery approaches that promote individual choice and self-determination. The importance and benefits of peer support and recovery approaches have been documented [33] and have been implemented increasingly across countries around the globe [1]. A hybrid model benefits service users in that during health system crises, such as a pandemic caused by an infectious disease when mental health needs are higher, access to mental health support can be maintained. Overall, this model offers promising potential as a vital resource to support the mental well-being of populations.

Using both models conjointly benefits not only service users and communities but also organizations that support mental health. By maintaining and strengthening both types of services, organizations that provide mental health services can build their capacities and be better prepared for sudden changes that might require suspending or limiting in-person services. This enhances flexibility and adaptability by maintaining a system that can dynamically switch between the two modalities.

Yet, despite the benefits of maintaining virtual services alongside in-person services, some PSWs and peers in our study reported a number of technology-related challenges that included difficulties obtaining internet connection or proper equipment, as well as limited skills with respect to the use of technology. Our findings are consistent with research which shows that providers and users of virtual mental health services report several limitations, such as difficulties with the adoption of the remote practice, and access and literacy challenges [11, 34–37]. Our findings also show that to be effective, a mental health support system that utilizes a virtual mode of service delivery requires appropriate technological tools and infrastructure, as well as appropriate support. In the case we studied, the organization advocated for and obtained access to the internet and equipment for peers. Further, the organization allocated extensive time to the training of PSWs and peers. PSWs, once versed on the use of the technology, offered help to peers in group settings and one-on-one when necessary. This kind of assistance and collaboration is common in peer support communities, where principles of mutuality and cooperation prevail, but this also suggests the importance of providing adequate resources to peer support communities so they can achieve their full potential.

Another challenge associated with the virtual environment is that computer-mediated communications provide fewer social context cues; hence individuals who join an online community may experience less personal connection [23]. This challenge was identified by some of our participants, prompting us to ask how a sense of community may be established and maintained when peers connect virtually.

### Sense of community

Ilioudi et al. (2012) refer to virtual communities in health care as “a group of people using telecommunication with the purposes of delivering health care and education, and/or providing support” [38, p.1]. These communities encompass a wide range of clinical services and technologies. During the COVID-19 pandemic, there was increasing attention to online recovery services and phone support, self-help and mental health self-management delivered virtually or in e-communities [39]. E-communities are critical for mental health support and have the potential to transform the philosophical approach to the provision of mental health services as they help bridge the gap between the high prevalence of mental health challenges and the relatively low capacity of mental health systems [40].

In peer support communities, individuals share experiential knowledge to encourage and pursue recovery as a mutual goal, showing common purpose and interdependence [41, 42]. Despite many peer support e-communities having been set up and having flourished during the COVID-19 pandemic and thereafter, there has been limited research on how the sense of community can be established or maintained in these groups. In studies of groups and communities more generally (and not only in the case of peer support), there has been focus on applying quantitative measurements and scales for the assessment of the sense of community, e.g., the Brief Sense of Community Index [43], and the Brief Sense of Community Scale [44]. These scales have been applied to study academic communities of practice [18], online education programs for different groups [45–46] and for individuals with serious mental illness living in community settings [47]. However, less research applies qualitative methods to explore in more depth this sense of community.

Literature shows that a sense of community is important in mental health support, especially during crises such as the COVID-19 pandemic [48]. A better understanding of the sense of community in virtual services could uncover factors that contribute to a positive therapeutic environment [49]. Our results identified five strategies to maintain a sense of community amongst peers and providers in a virtual environment during the COVID-19 pandemic. These findings highlight the importance of having a holistic and multidimensional perspective where the organization, providers, and peers all play a role.

The strategies we identified resonate with McMillan and Chavis’ conceptualization of a sense of community [20]. Their conceptualization highlights four elements: (a) membership (a feeling of belonging), (b) influence (a sense of mattering to the group), (c) integration and fulfillment of needs (a feeling that needs will be met through membership in the group), and (d) shared emotional connection (a belief that members have shared history and similar experiences). By “*acting collectively”* (as in our findings), individuals reinforce the notion that they belong to a community where their contributions matter and are valued. Acting collectively also allows the community to fulfill common needs. “*Building on organizational and peer culture*” involves recognizing the contributions of individual members that could reinforce the belief that each member has a meaningful impact on the community. This culture is inclusive and fosters integration and emotional connection among the members. “*Establishing multiple points of connection*” ensures that community members have diverse channels to interact, collaborate, and meet their needs. “*Maintaining a continuous presence and social interaction*” helps establish trust that membership in the community is a reliable path for meeting their needs. Finally, “*sharing lived experiences and learning together*” allows members to open up about their mental health (or other) challenges, contributing to an emerging collective narrative and shared history. Other organizations attempting to build or maintain a sense of community in a virtual space may find some of these strategies employed by the organization, the PSWs and the peers to be helpful.

### Limitations and directions for future research

Our study has a number of limitations. Concerns regarding security and privacy in virtual health care communities have been highlighted in research [10, 50]. Researchers have also pointed to potential conflicts within online communities set up for various purposes [51, 52]. Our paper did not examine these privacy and social concerns, however, evidence regarding these topics is important to provide guidance on how to make virtual spaces safe for peers who participate. Future research on these topics would be useful.

In addition, our findings pointed to peer support users who did not access the mental health support services when these transitioned to virtual platforms. We did not have access to these individuals, and it is not clear what factors contributed to their absence. Future research may explore whether and how technology-based boundaries become an impediment to seeking mental health support for some individuals. We also need a better understanding of the mental health of individuals who stopped using peer support when services moved online.

Our study focused on an organization and its members (PSWs and peers) and did not include in-depth attention to macro system level influences on or implications of peer support in a virtual space. The socio-economic aspects of adopting virtual work and services require further exploration including the financial return on investment and social returns (e.g. recovery) associated with using hybrid mental health support services. Overall, future research may identify and address system level influences that can hinder or facilitate mental health virtual services within community organizations, and how the needs of and services provided by these organizations may influence the allocation of resources and mental health indicators at a systems level.

### Implications for policy and practice

Our findings highlight the organization’s efforts to provide accessibility and support for both peers and PSWs and demonstrate the value of a proactive and responsive approach to addressing major change. Organizational and management support has been identified as a central factor in employees’ readiness when change occurs in an organization [53]. In fact, the COVID-19 pandemic situation highlighted the adaptability and resilience of peer support services and communities. As a manager in our study pointed out, the peer support organization was able to quickly and flexibly respond to the sudden surge in need for mental health support at a time when more institutionalized and strongly professionalized services were struggling to adapt. The resilience and adaptability of peer support organizations and programs are strengths in mental health care systems that are struggling to meet the needs of populations [1], yet these organizations and programs often receive a relatively small share of health care resources. Future policy may consider a more equitable allocation of resources to peer support services.

Another policy-related implication pertains to technology infrastructure and more specifically to who gets access to devices (such as smart phones and computers) and internet connections. Our study highlighted that lack of access to these resources was a boundary that challenged some peers seeking virtual support services. The peer support organization stepped in to create bridges by advocating with funders and tech providers. However, this leaves unsolved an issue that needs to be addressed at a higher societal level, namely the limited, yet necessary, resources available to some segments of the population (typically homeless individuals, people with disabilities, refugees and other groups). This issue should be an important consideration in future policy.

Finally, our study pointed to several practical implications based on the experience of the case we studied. For example, we pointed to the various strategies that peer organizations can use to maintain a sense of community in a virtual space. Further, in anticipation of the growth of virtual peer support services, organizations may consider the need for renewed training modules that integrate necessary skills relating to using technology for recovery support. Peer support organizations may also consider building their capacity to respond quickly to crises and major changes, as it is during these situations that their services may be in most demand.

## Conclusion

The important role of mental health community services and the changing drivers in mental health systems have been noted by researchers. Norton (2023) points out that “*mental health services are currently undergoing immense cultural, philosophical, and organizational change. One such mechanism involved in this change has been the recognition of lived experience as a knowledge subset in its own right*” [54, p.1]. The trends of peer support gaining in importance and being delivered in virtual as well as in-person spaces are poised to continue in the future. It is incumbent on researchers to continue studying the challenges and opportunities of peer support in its various models. Our study has been a step in this direction.

### Electronic supplementary material

Below is the link to the electronic supplementary material.

Supplementary Material 1

## Data Availability

The dataset used in this research is not publicly available as set out by the research ethics approval from the University of Ottawa and the consent forms signed by the participants. Further information is available from the corresponding author upon request.

## Abbreviations

**PSW**: Peer Support Worker
**UK**: United Kingdom
**US**: United States
**WHO**: World Health Organization
**REB**: Research Ethics Board
