# Supplementary material for: Transcending technology boundaries and maintaining sense of community in virtual mental health peer support: a qualitative study with service providers and users
Source: BMC Health Serv Res. 2024 Apr 24;24:510. doi: 10.1186/s12913-024-10943-y (PMC11040832; doi:10.1186/s12913-024-10943-y)
Supplement: Supplementary file 1 — Supplementary Material 1 [file 12913_2024_10943_MOESM1_ESM.docx]

**Transcending Technology Boundaries and Maintaining Sense of Community in Virtual Mental Health Peer Support: A Qualitative Study with Service Providers and Users**

**Interview Protocols**

[Questions for the Peer Support Workers 2](#_Toc157429018)

[Questions for the Peer Support Managers and Coordinators 3](#_Toc157429019)

[Questions for the Peer Support Service Users 6](#_Toc157429020)

[Closing Remarks for All Interviews 7](#_Toc157429021)

APPENDICES

Interview Guides

Questions for the Peer Support Workers

**Background Questions**

We will start with a few background questions about your engagement with peer support services.

1. Can you tell me briefly about your background in providing peer support services?
2. How long have you been with the peer support organization as a provider?
   - Have you been trained to provide peer support by the peer support organization?

**Experiences with Virtual Peer Support during the Pandemic**

Thank you. Now, I’d like to turn to some questions about your experiences of providing virtual peer support and your experiences with the pandemicave asked to better understand virtual peer support, and peer support during a pnademic?.

1. If I attended one of your typical peer support sessions during the pandemic, what would I see you doing?
2. What are the benefits of providing virtual peer support?
   - From your perspective, how did it help you and the service users during the pandemic?
3. What are the challenges that you faced delivering virtual peer support?
   - Regarding technology availability and knowhow, did you have any difficulties?
   - Make sure to probe here, e.g., about workspace at home.
4. How did you manage each of these challenges?
5. How has your role as a PSW been impacted because of virtual services?
6. Apart from the virtual services, how has your role as a PSW been impacted because of the pandemic?
7. How has virtual peer support impacted interactions? (between you and service users, among service users)
   - How has delivering virtual peer support changed your relationships with the service users?
8. Do you believe that the sense of peer support community has been impacted during the pandemic? If so, how?
9. How has virtual peer support impacted the boundaries that you maintain with service users?
10. How about the boundaries you maintain between personal and professional life?
11. Have you been able to maintain your own well-being during the pandemic?
    - (If yes): What are the factors that helped you maintain your well-being during the pandemic? (Probe about *supports* that were available **and** about *strategies* interviewee has used.)
    - (If not): Can you tell me about your experiences?
12. Did you have access to a community of PSWs? How did this influence you?

**Supports**

1. Did you follow any training on virtual peer support? If so, what was this training?
   - In what ways was the training helpful or unhelpful?
2. (If not addressed before): What other supports were available?
3. What supports were missing?
4. What suggestions or recommendations do you have to managers and to policy makers regarding peer support during a pandemic? Let’s start with managers.
   - What about suggestions or recommendations to policy makers?

Questions for the Peer Support Managers and Coordinators

**Background Questions**

We will start with a few background questions about your engagement with peer support services.

1. How long have you been with the peer support organization and what are your current role and responsibilities?
2. Can you tell me briefly about your background as peer support manager/coordinator?
3. How do you work with other managers, coordinators or PSWs? In other words, how is your role defined in relation to others? How do you interact with others?
4. How does one become a manager (or coordinator) in this peer support organization?
   - Does that involve any training? (if yes): can you tell me briefly about the training you received?

**Experiences with Virtual Peer Support during the Pandemic**

Thank you. Now, let’s talk about your experiences with the virtual peer support during the pandemic.

1. How did the pandemic influence how you perform your role as manager (or coordinator)?
2. Can you describe the process of shifting from in-person to virtual form in the early stages of the pandemic?
3. What were the challenges?
   - How did you experience these changes?
   - What strategies did you adopt to minimize the challenges?
4. How did you prepare yourself and PSWs for virtual services?
5. Do you think that virtual services facilitated providing support to the service users? How?
   - Make sure to probe if there are new service users and why that is the case.
6. In your opinion, what are some opportunities associated with the pandemic?
   - Make sure to probe on the opportunities associated with virtual peer support
   - How do you think maintaining peer support virtually has helped the peer community during the pandemic?
7. Which of the changes you have made during the pandemic should be sustained and why?

**Experiences with the Organizational Factors**

Thank you. Now, I’d like to ask you about how contextual/organizational factors have influenced operations during the pandemic:

1. How do you think the culture of the organization as a peer support organization has influenced the shifts in work that have occurred due to the pandemic?
2. What measures has the peer support organization adopted to help managers (or coordinators) during the pandemic?
   - Make sure to probe on e.g., training, and equipment for virtual work.
3. What are some of the strengths and weaknesses of the present team of managers, coordinators and PSWs that you think has impacted the work of the peer support organization during the pandemic?
   - Let’s start with the strengths.

- Make sure to probe on the skills and strategies of the team.

1. What suggestions or recommendations do you have to other managers of peer support and to policy makers regarding peer support during a pandemic?
   - Let’s start with managers.
   - How about suggestions or recommendations to policy makers?

Questions for the Peer Support Service Users

**Background Questions**

We will start with a few background questions about your engagement with peer support services.

1. Can you tell me briefly about your background in receiving peer support services?
2. How long you have been attending peer support sessions, and have you been receiving one-on-one peer support or group peer support?

**For service users who *did not continue* peer support during the pandemic**

1. Can you explain the reasons behind not continuing to receive peer support after the transition to a virtual platform?
   - Make sure to probe about the preferences, concerns, confidentiality consideration, technical literacy, and perceptions of what effective peer support is.
2. How has not receiving peer support impacted you?
   - Make sure to probe about other supports or coping mechanisms

**For service users who *have started* receiving peer support during the pandemic**

1. Have you ever had the experience of receiving peer support before?
   - If not, what is it about the pandemic or virtual services that encouraged you to access these services?

**For service users who *have continued* using peer support:**

1. How have your experiences with peer support changed during the pandemic?
   - How do you compare your experience with the in-person services?

**For service users who *have started or continued* using peer support:**

1. Would you feel that you receive the support that you need through the virtual peer support sessions?
2. How would you describe your relationship with the PSWs in these virtual meetings?
   - How was it helpful for you?
   - Have you had any challenges in your relationships with the PSWs and if so, what are they?
3. What challenges did you have in accessing virtual peer support services?
4. Make sure to probe about the preferences, concerns, confidentiality consideration, technical literacy, and perceptions of what effective peer support is.
5. How would you describe your relationship with other service users in virtual meetings?
   - Have you had any challenges in your relationships with other service users and if so, what are they?
6. Do you believe that virtual services should be continued? If yes, why and if not, why not?
7. What issues would you like the peer support organization to consider when designing services *during the pandemic*?
8. What issues would you like the peer support organization to consider when designing services *once the pandemic is over*?
9. What other suggestions do you have?

Closing Remarks for All Interviews

**Demographic Questions**

Thank you. That was the last question, and now I have a few demographic questions.

1. How would you describe your gender identification?
2. Would you say your age is between 20-29, 30-39, 40-49, 50-59, 60-69, or over 70?
3. How would you describe your ethnicity?

**Closing Questions**

Thank you. Now there’s a chance for us to make sure we haven’t missed anything important in our discussion.

1. Is there anything you would like to add?
2. Is there anything I didn't ask that I should have asked?
3. If I have any other questions or need additional clarifications, is it ok for me to contact you again?

**Closing Thanks**

That was my last question. Thank you for your time and for participating in this interview.
